# Orthogeriatrics and Hip Fracture Care in the UK: Factors Driving Change to More Integrated Models of Care

**DOI:** 10.3390/geriatrics3030055

**Published:** 2018-08-28

**Authors:** Mark Middleton

**Affiliations:** Department of Trauma and Orthopaedics, St George’s University Hospitals NHS Foundation Trust, Blackshaw Road, Tooting, London SW17 0QT, UK; m.c.f.middleton@doctors.org.uk; Tel.: +44-020-8672-1255

**Keywords:** hip fracture, geriatric care models, orthogeriatric care, national hip fracture database

## Abstract

In the United Kingdom (UK), approximately 80,000 hip fractures each year result in an estimated annual cost of two billion pounds in direct healthcare costs alone. Various models of care exist for collaboration between orthopaedic surgeons and geriatricians in response to the complex medical, rehabilitation, and social needs of this patient group. Mounting evidence suggests that more integrated models of orthogeriatric care result in superior quality of care indicators and clinical outcomes. Clinical governance through national guidelines, audit through the National Hip Fracture Database (NHFD), and financial incentives through the Best Practice Tariff (providing a £1335 bonus for each patient) have driven hip fracture care in the UK forward. The demanded improvement in quality indicators has increased the popularity of collaborative care models and particularly integrated orthogeriatric services. A significant fall in 30-day mortality has resulted nationally. Ongoing data collection by the NHFD will lead to greater understanding of the impact of all elements of hip fracture care including models of orthogeriatrics.

## 1. Introduction

Worldwide projections demonstrate the increasing importance of hip fractures [1], which, combined with challenging comorbidities represent an ever-growing burden to society [2]. In the United Kingdom (UK), approximately 80,000 hip fractures each year result in an estimated annual cost of two billion pounds in direct healthcare costs alone [3]. The initial collaboration between geriatric medicine and orthopaedics [4] has evolved into a subspecialty of orthogeriatrics as a response to the complex medical, rehabilitation, and social needs of this patient group. National guidelines have been published recommending increased co-operation between orthopaedic surgeons and geriatricians in the UK National Health Service (NHS) [5,6,7]. As a result, the UK has led the way [8] in defining the now-accepted standard of early orthogeriatric collaboration [9]. This approach to modern hip fracture management should achieve the goals of co-ordinated multidisciplinary care, early surgery, and facilitated discharge [10].

## 2. Orthogeriatric Models of Hip Fracture Care

Five broad models of hip fracture care have been defined (summarized in Table 1) [6,11], though of course there are many variations within them. The first category of orthopaedic care with geriatrician consultation when requested has been termed “traditional care” [6]. This sits alongside the geriatric orthopaedic rehabilitation unit model where the patient undergoes standard orthopaedic peri-operative care before transfer for rehabilitation under geriatricians. The remaining three models involve collaboration between the specialties and could therefore be defined as orthogeriatric models of hip fracture care [12]. The orthogeriatric liaison or routine review model is defined as care taking place within an orthopedic ward with consistent geriatrician consultation. The opposite model is also proposed, with the primary responsibility of care under geriatricians with orthopaedic consultation. The final model is that of shared or combined care, also known as an integrated hip fracture service or co-management [6,12]. This integrated care model sees the patient treated on a unit where both the orthopaedic surgeon and geriatrician share responsibility for their care [13,14].

Studies of orthogeriatric care have reported mixed efficacy, possibly due to the wide variety of clinical models of care employed [15,16,17,18]. A review by Kammerlander et al. [19] and Grigoryan et al.’s meta-analysis [12] provide strong evidence supporting improved outcomes with orthogeriatric collaboration. They are unable to determine which model is best, but do note a trend towards use of more integrated models in later studies [19]. Subsequent evidence suggests that more integrated care is better in terms of lower mortality and quality of care indicators [20], such as length of stay [21,22,23,24,25]. Studies which directly compare different models of orthogeriatric care have found the integrated service model to be superior to a routine review model in terms of mortality, time to surgery and length of stay [18,26]. A recent systematic review [27] of 18 eligible studies found that reduced mortality was associated with the term “orthogeriatric ward”. As further evidence mounts that integration of services results in better outcomes, this will drive change towards the use of such shared care models.

## 3. Clinical Governance, Incentives, and Finances

The Department of Health defines clinical governance as “a framework through which NHS organisations are accountable for continuously improving the quality of their services and safeguarding high standards of care” [28]. With recognition that across the NHS there is heterogeneous infrastructure and care, with wide variation in management and resources, these principles have been rigorously applied to hip fracture care. This has been achieved through national guidelines and quality statements [5,6,29], audit through the National Hip Fracture Database (NHFD) [30], and financial incentives through the best practice tariff (BPT).

The NHFD was launched in 2007 with the explicit aim of using the synergy of clinical standards, audit and feedback to improve hip fracture quality of care and outcomes [31]. It has since been used to benchmark quality indicators and guide the funding of best practice (BPT) in the NHS [30]. Providers can use the data for audit purposes and compare themselves with other units. National benchmarking data can identify outliers and give the necessary leverage to obtain the resources required to change practice [18]. Mandatory collection and submission of data to achieve the BPT has effectively allowed it to become one of the largest prospective clinical audits taking place in the UK. Clear evaluation and transparent reporting of this data enables the spread of good practice and drives up hip fracture care standards [32]. Improved productivity can be the result of monitoring itself [2] and benefits have been demonstrated by implementing the NHFD [33]. Annual reports demonstrate successive improvements in audited standards of care as well as a steady fall in mortality in the time that it has been operational [30].

The BPT was introduced in 2010 and expanded in 2011, offering hospitals a £1335 “bonus” payment per hip fracture patient that is managed according to a set of quality indicators collected on the NHFD [30]. These include performing surgery within 36 h of admission, orthogeriatrician review within 72 h of admission, evidence of multidisciplinary care in the acute phase, and secondary fracture prevention [34]. Quality improvement projects in response to BPT have reduced the time to surgery of hip fracture patients by introducing additional trauma operating sessions so that theatre time is available to meet demand [35,36]. Oakley et al. [34] demonstrated that mortality was reduced in cases where BPT was achieved. The financial incentives provided have resulted in units searching for the most efficient methods of achieving the quality of care parameters. Thus, an increasing number of hospitals have adopted interprofessional orthogeriatric models of care [32] and compliance with BPT has risen from approximately 40% to 60% of all hip fracture patients over five (5) years [37].

Organisational factors have also played a role in further integration of services having risen to the forefront as units attempt to attain the BPT. The role of an orthogeriatrician is clinical, but also requires involvement in system change, service development and leadership [38]. The implementation of a hip fracture programme has been shown to significantly increase the probability of earlier surgery [39], whilst delays caused by anticoagulants can be mitigated by a multidisciplinary team [40]. High- risk, high-need patients will be identified earlier and optimized. Osteoporosis care to reduce the risk of secondary fractures can be poorly co-ordinated [41], but the efficiency of this care is improved by an orthogeriatric fracture unit [42,43]. Interestingly, hip fracture care in the UK does not suffer from the apparent “weekend effect” where higher observed mortality rates are attributed to lower staffing levels in place at weekends. This is due to infrastructures instigated to achieve BPT [44,45].

In times of financial austerity, the role of BPT incentives in persuading service change should not be underestimated. In the UK, improved compliance with BPT can also provide the financial opportunity to invest in and further develop services. However, even without the inducement of BPT payments outside of the UK, integrated services have been found to be more cost-effective in moderate volume units and are cost-saving in high-volume units [46]. The majority of these effects appear to be due to a reduction in length of hospital stay [47,48,49], but expediting the time to surgical treatment also contributes [50].

## 4. The Present and the Future

A national organization survey found an increase in orthogeriatrician hours from 1.5 to 4 h per patient over the period from 2010 to 2014 [38]. This corresponded to higher recorded rates of prompt surgery and a relative reduction of 3.4% in 30-day mortality. [38]. Progress in collaboration between orthopaedic and geriatric specialties is also recorded in the NHFD facilities audit data [37]. More than 80% in recent years have described their unit’s model of orthogeriatric care as “shared care” or “routine review”, with only 4% in 2017 describing a traditional model. In more recent years, more have described shared care than routine review demonstrating a move towards further integrated models of care. Evidence continues to be published from units within the UK where integrated services have resulted in improvements of care [18,51], providing ideas that could be adopted at other institutions.

The improvements in outcome and quality of care indicators have been remarkable in recent years. The NHFD has demonstrated a steady reduction in 30-day mortality over 10 years from 10.9% in 2007 to 6.7% in 2016 [30]. This may well reduce further if the room for improvement in BPT compliance is realized. However, further challenges are expected from increasing numbers of patients with a projected rise to 100,000 hip fractures per year and inflation adjusted costs of £3.6–5.6 billion by 2033 [52]. The NHS will aim to provide the best possible care as efficiently as possible. Information recorded by the NHFD encompasses all aspects of intra-operative and post-operative care, including collaboration with disciplines such as anaesthesia, physiotherapy and social care [30]. Therefore, the ongoing data collection will lead to greater understanding of these aspects of hip fracture care as well as orthogeriatric models. More research into quality of life and functional recovery rather than just clinical care measures and mortality have also been targeted [10,12].

## 5. Conclusions

The subspecialty of orthogeriatrics has developed in response to clinical, social, and financial needs. There is a mounting body of evidence that the more integrated models of care result in improvements in both quality indicators and outcomes. Such collaborative care appears to be increasing in popularity in the UK in response to clinical governance, national audit data and financial incentives. A comparable expansion of orthogeriatrics may be less likely in other healthcare systems due to workforce availability [38], though adoption of governance and models of care from the UK experience may be beneficial.

## Figures and Tables

**Table 1:** Hip fracture care models.

Model	Description
Traditional care	Orthopaedic care with geriatrician consultation on request
Geriatric orthopaedic rehabilitation unit	Orthopaedic care until transfer for rehab under geriatricians
Orthogeriatric routine review	Orthopaedic ward with consistent geriatrician consultation
Orthopaedic routine review	Geriatric ward with consistent orthopaedic consultation
Integrated hip fracture service (Co-management)	Shared responsibility between orthopaedics and geriatricians
